# A cross-sectional study of mental health and suicidality among trans women in São Paulo, Brazil

**DOI:** 10.1186/s12888-021-03557-9

**Published:** 2021-11-10

**Authors:** Arianne Reis, Sandro Sperandei, Paula Galdino Cardin de Carvalho, Thiago Félix Pinheiro, Ferdinando Diniz de Moura, José Luis Gomez, Patrícia Porchat, Francisco Inácio Bastos, Willi McFarland, Erin C. Wilson, Maria Amélia Veras

**Affiliations:** 1 School of Health Sciences, Western Sydney University, Penrith, Australia; 2 Translational Health Research Institute, Western Sydney University, Penrith, Australia; 3 Faculdade de Ciências Médicas, Santa Casa de São Paulo, São Paulo, Brazil; 4 School of Sciences, São Paulo State University, Bauru, Brazil; 5 Instituto de Comunicação e Informação Científica e Tecnológica em Saúde, Fundação Oswaldo Cruz, Rio de Janeiro, Brazil; 6 San Francisco Department of Public Health, Center for Public Health Research, San Francisco, CA USA; 7 Department of Epidemiology and Statistics, University of California, San Francisco, CA USA; 8 San Francisco Department of Public Health, Trans Research unit for Equity (TRUE), San Francisco, CA USA

**Keywords:** Transgender women, Mental health, Suicidal ideation, Suicide attempt, Risk factors

## Abstract

**Background:**

Trans women have been shown to experience disproportionately poor outcomes in physical and mental health. Although it is common to talk about the violence against trans people, little is still known about mental health outcomes and experiences of suicidality among trans women, particularly in developing countries. This study aims to investigate risk factors and associations with mental health, suicide ideation and suicide attempts among trans women in the largest metropolitan area in Brazil.

**Methods:**

Trans women living in São Paulo were recruited between May 2017 and July 2019 using the long-chain peer referral method Respondent-Driven Sampling. Multivariate regression models were used to investigate the associations with K10 score classification (logistic) and suicidal ideation/suicide attempt (ordinal logistic).

**Results:**

A total of 763 trans women were included in the study. Over one quarter (26.5%) of trans women had been diagnosed with anxiety in the past, and close to one in five (19.1%) trans women had received a diagnosis of depression. More than two in five (41.9%) trans women had moderate to severe psychological distress. More than half of all participating trans women reported having previously either experienced suicidal ideation or attempted to take their own lives (25.0 and 31.2% respectively). In multivariate regression, moderate to severe psychological distress was associated with homelessness, income, current sex work, use of stimulant drugs, history of physical abuse, depression diagnosis and access to mental health treatment. Suicidal ideation and suicide attempts were associated with race/skin color, living arrangements, marital status, current sex work, history of sexual violence, depression and PTSD diagnoses, access to mental health treatment and psychological distress.

**Conclusions:**

This study showed that there is a significant association between mental health conditions, lack of treatment for these conditions and suicidality among trans gender women. Findings point to the need for a structural transformation in Brazil that enables a reduction in the social inequality and violence that impact the mental health of trans women. A number of recommendations to achieve this are provided.

## Background

The role of mental health in achieving global development goals and maintaining the stability of the social fabric is particularly noticeable when high rates of depression, anxiety disorders, suicidality, and other mental disorders affect the economic and social functioning of groups, institutions, and nations [1–3]. For example, suicide is estimated to contribute about 1.5% of the global burden of disease and is the third leading cause of death among youth in Brazil [4–6]. Despite an increasing awareness of the burden of mental health in society, investment in mental health support within public health systems is still largely inadequate. This is particularly the case in low and middle-income countries, where public investment in mental health care is low. For instance, while France spends approximately US$420 per person in mental health, and Australia spends USD$265, Brazil spends only USD$1.79 [7].

Apart from the low investment in mental health care, in Brazil medicalization stands out as the main form of intervention and treatment of the symptoms presented in common mental health disorders, with little acknowledgement of the particular conditions of vulnerability of patients, who often belong to population groups marked by specific types of violence, such as gender violence or racial discrimination [8, 9]. Local studies point out a high correlation between the emergence of these disorders and socioeconomic conditions such as gender, race, poverty and low education level [10, 11]. Whether such diagnoses point to clinical conditions or a medicalization of social issues is still not clear. In any case, fighting the symptom through medication is not likely to eliminate its structural determinants.

When considering vulnerable populations, trans people report high rates of mental health concerns, such as depression, resulting in higher prevalence of suicidality (i.e. suicidal thoughts, suicide attempts and suicide rates) compared to the cisgender population [12]. Recent evidence suggests that approximately 40% or more of trans people have attempted suicide at least once in their lifetime [13]. Within this population, studies show that trans women have a higher prevalence of suicidal ideation compared to trans males, but there is no conclusive evidence regarding the predominance of suicide attempts [14].

Suicidality and other mental health issues may be precipitated by intolerance and violence related to gender identity that trans women face [15]. Family rejection and mistreatment, harassment, verbal and physical violence, sexual assault at workplace or schools are frequent among trans women, while other discriminatory situations and human rights violations also experienced by trans women include when accessing medical care, housing, and they are often targeted or assaulted by police officers [16, 17]. Magno, Dourado [18] have reported high levels of gender-based discrimination and a banalization of this violence against trans women, fostered by the pervasive “machismo” (male chauvinism), misogyny, and patriarchy sentiments entrenched in the society. Experiences of discrimination perpetrated by the family have severe emotional impact and often initiate a trajectory of displacement and economic vulnerability that push many trans women into sex work. In the context of sex work, trans women suffer diverse types of aggression, particularly sexual violence [18].

Trans women have also been shown to experience disproportionately poor outcomes in physical and mental health. Although national data on HIV prevalence among trans women are not available in Brazil due to the lack of information about gender identity in case reports, local studies suggest that they constitute the segment most affected by HIV [19, 20]. Commonly reported determinants of poor psychological wellbeing for trans women in Brazil are associated with the lack of a fixed address, lower levels of education, verbal and sexual violence, and dissatisfaction with personal relationships, support from friends or availability of gender-affirming surgeries and procedures [21]. High levels of depression and problematic use of drugs and alcohol in Brazilian trans women [22] follow the tendency of trans people being burdened by mental health concerns in other countries [23].

Although it is common to talk about the violence against trans people, especially considering that Brazil is the record holder in the murder of trans people [24], little is known about mental health outcomes and experiences of suicidalit y among trans women in Brazil [25–27]. In a country where discrimination based on gender is high, human rights violations are common and lack of investment in mental health is prevalent, it is important to learn more about the experiences of mental health and suicidality among trans women to explore risk factors and establish potential areas of intervention. This exploratory study aims to investigate factors associated with mental health conditions, suicide ideation and suicide attempts among trans women in Brazil.

## Methods

### Study design

Data presented here are from the São Paulo TransNational Study, one of four sites included in an international collaborative research project with trans women. Other sites included San Francisco (USA), Nanjing (China) and Asunción (Paraguay). São Paulo is the largest metropolis in Brazil, located in the southeast region of the country and hosting a population of over 12 million people.

Trans women were recruited between May 2017 and July 2019 into the cohort using the long-chain peer referral method Respondent-Driven Sampling (RDS) [28]. They were invited to participate in the survey during the formative phase of the study, which included four focus groups (including an average of 8 participants in each) and eight in-depth interviews. Participants from this stage of the study were recruited through community-based organizations and community leaders based in the central region of the city of São Paulo and in two highly populated peripheral regions, South Zone and East Zone. Nine of these first participants were then encouraged, via referral coupons (3–5 per women), to invite other eligible trans women from their social networks to participate in the study. The selection of the nine ‘seeds’ intended to capture a diversity of participants living in different regions, working in different sectors and with different levels of education. Those who were recruited by the ‘seeds’ were then asked to refer other participants from their social networks and so on. Eligibility criteria included: 1) being assigned male sex at birth and now identifying as a trans woman, woman, ‘travesti’ or other gender identity on the female spectrum; 2) being aged 18 years and older; 3) being in possession of a referral coupon (for those being the first wave of participants identified by the research team); and 4) being able to provide informed consent. Participants completed structured interviews and were tested for HIV, although the latter is not included in the material presented in this article. Participants were given R$30.00 (~USD$10) for completing the survey and HIV testing at their initial visit. Participants were also given R$30 for each eligible referral to the study. Referrals to HIV care were made for trans women testing positive. Referrals to other health care and prevention programs, including PrEP to HIV-negative trans women, were offered at each visit.

### Data collection

Trans women were interviewed face-to-face by trained staff using computers to record answers. Interviews lasted, on average, 45 min, and were conducted at the research department of the Centro de Referência e Treinamento em DST/AIDS.

The data collection instrument was a comprehensive socio-behavioral questionnaire comprised of questions about sociodemographic and behavioral characteristics, including use of alcohol and drugs, and experiences of homelessness, as well as experiences of stigma, discrimination, abuse and violence, and health-related issues, including access to and use of mental health-related care. Psychological distress was assessed using the 10-item Kessler Psychological Distress Scale (K10), which categorizes psychological distress into four levels: 1) well or generally good mental health (for scores ≤20); 2) potentially mild psychological distress or mental health disorder (for scores 20–24); 3) potentially moderate psychological distress or mental health disorder (for scores 25–29); and 4) potentially severe psychological distress or mental health disorder (for scores > 29). Participants were asked whether they had ever been diagnosed with anxiety, depression or post-traumatic stress disorder (PTSD), and whether they had ever had suicidal ideation or attempted suicide. Measures of discrimination, abuse and violence included experiencing verbal abuse or harassment due to their gender identity, race/skin color; experiencing physical abuse, harassment or violence due to their gender identity, race/skin color; and experiencing sexual violence due to their gender identity.

### Ethical considerations

The protocol was reviewed and approved by the Ethical Committee of the Centro de Referência e Treinamento em DST/AIDS and by the Brazilian National Ethics Review Board (CONEP-CNS, #1880217), and the University of California San Francisco IRB (#15–17,775). All participants provided written informed consent.

### Data treatment and analysis

Given the exploratory nature of this study, we considered a number of potential explanatory variables for the data analysis process: age, sex orientation, level of education, race/skin color, homelessness before age 18, homelessness after age 18, living situation, marital status, monthly income, receipt of government financial assistance, engagement in sex work, alcohol and drug use, access to treatment of mental health conditions, depression, PTSD and anxiety diagnoses, psychological distress based on K10 results, suicidal ideation in lifetime, suicide attempt in lifetime, and experiences of violence and discrimination.

Some variables were re-coded to avoid categories with very low frequencies and to improve interpretation of results. First, the age of participants, included in the model as a continuous variable, was re-coded to start with the minimum age (i.e. 18 years old) as zero. This was done so that the intercept for any model that includes age could be interpreted as being related to the minimum age of the cohort. It is important to clarify that this change only affects the intercept of the model and has no effect on odds-ratio.

For the variable ‘sex orientation’, we excluded very low frequency categories (i.e. queer, questioning/in doubt, asexual, and other), as it did seem reasonable to combine them with any other category and their extremely low frequency made it impossible to get estimates for them.

The variable ‘living arrangements’ was re-grouped based on what was considered the stability of the arrangements, with those who owned or rented a house being classified as living in a ‘stable’ condition, and those living on the streets, couch-surfing, in shared rooms or in institutions being classified as living in ‘unstable’ conditions.

Monthly income was categorized according to the minimum wage in Brazil at the time of the survey (R$937 or ~ US$ 283), and group in less than one, one to two, and more than 2 minimum wages per month.

Those who reported having employment as a source of income were classified as ‘employed’, irrespective of other sources. Those reporting any kind of social support or pension from the government (excluding retirement pension but including permanent sickness pension) were classified as under social assistance.

A ‘sex work’ variable was obtained from the combination of different variables. Those reporting no history of any kind of financial benefit (i.e. money, goods or a place to live) from having sex were classified as ‘never’. Those reporting having received benefits in the past but not currently were classified as ‘past only’. Finally, those reporting current income from sex work were divided into those who reported other sources of income and those who had sex work as their sole source of income.

The use of alcohol in the past 12 months was categorized into: ‘no use of alcohol’, ‘use of alcohol but not before or during sex’, and ‘use of alcohol including before or during sex’ in cases where they reported use before or during sexual intercourse, regardless of frequency (i.e. if they reported using alcohol at least once in the past 12 months they were considered users). Drug use was treated in a similar way. However, sub-groups of drugs were created. The ‘cannabic drugs’ group included the use of marijuana in natura or in a synthetic form. The ‘stimulant drugs’ group included methamphetamine in any form, crack and cocaine. Then, an ‘other drugs’ group was created to include the remaining types of drugs investigated (i.e. ecstasy, heroin, pain killers, ketamine, solvents, poppers and others).

The K10 score was dichotomized as ‘none/mild’ (scores ≤29) and ‘moderate/severe’ (scores 30–50). Combining K10 scores in two groups was to ensure sufficient numbers of cases, and also reflects a high likelihood that a significant proportion of participants in the two high categories would have a diagnosable mental disorder. Normative data on the K10 indicates a 12-month prevalence of 55% for any ICD-10 mental disorder among those who are classified in this measure as having ‘moderate’ and ‘severe’ scores [29].

For the modelling process, the RDS sampling structure was not considered, as recent evidence suggests that RDS data can be modelled without taking into consideration the RDS structure of data collection [30, 31]. Initially, bivariate logistic models were used to observe the relationship between variables and the two K10 classes. Then, a stepwise regression approach was used to obtain the most parsimonious model. Given the exploratory nature of the study, the multivariate modelling process commenced with a full model, containing all potential explanatory variables, using the stepwise regression as a tool to achieve a final model containing the minimum number of variables without losing significant information. The results are presented as odds-ratios (OR) and 95% confidence intervals.

To model lifetime suicidal ideation and suicide attempt behaviors, the two respective questions were combined as a three-level ordinal variable, containing those who never ideated or attempted suicide (‘none’), those who ideated but had not attempted (‘ideation’), and those who attempted suicide at least once in their lifetime (‘attempt’). This outcome variable was modelled using an ordinal logistic regression model also including all potential explanatory variables, starting with a complete model using stepwise regression to obtain the most parsimonious model. In addition, the K10 score was entered as an exposure for this model but using its original four-level categorical form. The parallel regression assumption of the model was tested using the Brant test [32].

The results of the multivariate models were used to create high and low risk profiles based on the combination of identified high and low risk characteristics. For each profile, the likelihood of suicidal ideation and suicide attempt was estimated based on the coefficients derived from the model for the identified characteristics.

## Results

### Characteristics of participants

Out of 792 completed surveys, 29 (3.7%) presented missing data in at least one of the variables considered in the study and were therefore excluded from the analysis. No clear pattern was observed in the missing information and no significant difference between excluded and included participants was found.

The demographic characteristics of the sample are presented in Table 1. Participants were 30 ± 9 years of age on average. Most participants reported their sexual orientation as straight/heterosexual (81.5%), identified themselves as Black or ‘Parda’ (mixed-race) (70.1%) and were single (80.2%). Levels of education were generally low, with only 9.6% of participants going beyond high school. Over one quarter of the participants had been homeless as a child or teenager (26.6%) and well over one third had been homeless as an adult (39.4%). Over 40% of trans women participants reported living in unstable conditions, that is, not living in a rented or owned home. In addition, almost half of participants (46.7%) lived on a monthly income of up to one minimum wage (R$ 937 or the equivalent to ~US$283 per month), and only 20.2% received some form of financial social assistance from the government. Only 15.9% of all trans women participants reported never working as a sex professional in the past.

**Table 1:** Demographic characteristics of trans women participants and correlates of psychological distress, suicidal ideation and suicide attempt (*N* = 763)

Demographic characteristics	Descriptive Frequency		Bivariate Models			
			K10 Score		Ideation/Attempt	
	Mean or N	SD or %	Estimate	95%CI	Estimate	95%CI
Age (years)	30	9	0.98	0.968–1	1.01	0.995–1.025
18–24	255	33.3	1	–	1	–
25–44	450	58.8	1.23	0.903–1.686	1.18	0.886–1.567
45–65	60	7.8	0.55	0.285–1.001	1.39	0.824–2.326
Sex Orientation						
Straight/heterosexual	622	81.5	1	–	1	–
Gay/lesbian	75	9.8	1.28	0.791–2.078	0.8	0.505–1.247
Bisexual	54	7.1	1.47	0.839–2.57	0.99	0.593–1.655
Pansexual	12	1.6	1.47	0.455–4.743	3.25	1.112–10.75
Education						
Primary school	302	39.6	1.76	1.04–3.062	0.89	0.574–1.396
Middle school or high school	388	50.9	1.37	0.817–2.362	0.77	0.502–1.195
Technical or university degree or more	73	9.6	1	–	1	–
Race/Skin Color						
White	201	26.3	1	–	1	–
Black or Parda (mixed race)	535	70.1	1.36	0.98–1.911	0.7	0.516–0.938
Other	27	3.5	0.86	0.352–1.964	0.79	0.369–1.677
Homeless before age 18 years	203	26.6	1.5	1.086–2.074	1.51	1.116–2.038
Homeless after age 18 years	301	39.4	1.87	1.391–2.512	1.96	1.495–2.582
Living situation						
Own a house or apartment	139	18.2	1	–	1	–
Rent a house or apartment	313	41	1.06	0.697–1.613	0.83	0.574–1.214
Couch surfing with friends or family	63	8.3	2.22	1.215–4.1	1.71	0.987–2.96
Homeless/shelter	153	20.1	2.37	1.482–3.823	2.38	1.542–3.683
Other	95	12.5	1.44	0.842–2.463	1.17	0.725–1.881
Marital status						
Single	612	80.2	1	–	1	–
Married/de facto	127	16.6	0.9	0.608–1.331	1.5	1.054–2.127
Divorced or separated or widowed	24	3.1	1.95	0.857–4.582	1.56	0.722–3.401
Monthly income in minimum wages (R$ 937 or approx. US$ 283/month)						
Up to 1 minimum wage	356	46.7	1	–	1	–
1 to 2 minimum wages	230	30.1	0.75	0.534–1.048	0.82	0.604–1.118
More than 2 minimum wages	177	23.2	0.63	0.435–0.915	0.74	0.527–1.035
Recipient of financial social assistance from government	154	20.2	1.08	0.757–1.547	1.7	1.229–2.366
Engagement in sex work						
Never	121	15.9	1	–	1	–
In the past only	260	34.1	1.31	0.834–2.069	1.29	0.871–1.923
Currently (partial source of income)	126	16.5	1.84	1.103–3.097	1.21	0.765–1.909
Currently (sole source of income)	256	33.6	1.73	1.108–2.737	0.63	0.424–0.944

### Experiences of alcohol and drug use

Drug and alcohol use both generally and during sex was common among participants (Table 2). Close to three quarters of participants (72.7%) reported using alcohol at least once in the past year, with over half (53.6%) reporting using it before or during sex. Use of cannabic drugs in the past year was similar to the use of stimulant drugs, such as cocaine and crack. In both cases, around one third of participants had used one of the identified drugs in the past 12 months, including before or during sex (36.4% cannabic drugs and 32.9% stimulant drugs). Other types of drugs were less favored during sex (10.2%) but were comparatively more commonly used outside of sex experiences (26.1%).

**Table 2:** Alcohol and drug use of trans women participants in the past 12 months and correlates of psychological distress, suicidal ideation and suicide attempt (*N* = 763)

Alcohol and Drug Use Characteristics	Descriptive Frequency		Bivariate Models			
			K10 Score		Ideation/Attempt	
	N	%	Estimate	95%CI	Estimate	95%CI
Use of alcohol in the past 12 months						
No use of alcohol	208	27.3	1	–	1	–
Use of alcohol but not before or during sex	146	19.1	0.71	0.454–1.1	0.65	0.439–0.969
Use of alcohol including before or during sex	409	53.6	1.22	0.87–1.712	0.91	0.668–1.246
Use of cannabic drugs in the past 12 months						
No use of cannabic drugs	406	53.2	1	–	1	–
Use of cannabic drugs but not before or during sex	79	10.4	1.41	0.867–2.29	0.86	0.537–1.358
Use of cannabic drugs including before or during sex	278	36.4	1.17	0.86–1.598	1.26	0.95–1.672
Use of stimulant drugs in the past 12 months						
No use of stimulant drugs	424	55.6	1	–	1	–
Use of stimulant drugs but not before or during sex	88	11.5	2.12	1.332–3.375	1.05	0.68–1.611
Use of stimulant drugs including before or during sex	251	32.9	1.8	1.312–2.478	1.53	1.149–2.051
Use of other drugs in the past 12 months						
No use of other drugs	486	63.7	1	–	1	–
Use of other drugs but not before or during sex	199	26.1	1.43	1.026–1.997	1.18	0.864–1.598
Use of other drugs including before or during sex	78	10.2	1.6	0.988–2.588	1.51	0.975–2.33

### Experiences of discrimination and violence

Verbal and physical abuse as well as sexual violence were experienced by high proportions of trans women participants (Table 3). More than four in five trans women had been verbally abused in the past (87.4%), and close to two thirds had experienced physical abuse (62.3%). More than one in five trans women had experienced sexual violence at least once in the past (20.2%).

**Table 3:** Abuse and violence experienced by trans women participants and correlates of psychological distress, suicidal ideation and suicide attempt (*N* = 763)

Variables	Descriptive Frequency		Bivariate Models			
			K10 Score		Ideation/Attempt	
	N	%	Estimate	95%CI	Estimate	95%CI
Have experienced verbal abuse at least once in the past	667	87.4	1.79	1.138–2.874	1.96	1.292–3.006
Have experienced physical abuse at least once in the past	475	62.3	1.7	1.258–2.309	1.86	1.411–2.46
Have experienced sexual violence at least once in the past	154	20.2	1.36	0.956–1.947	2.24	1.607–3.141

### Mental health diagnosis and services received by participants

Over one quarter of trans women had been diagnosed with anxiety in the past (26.5%), and close to one in five trans women had received a diagnosis of depression (19.1%) (Table 4). Post-traumatic stress disorder was less frequently reported, with 5.4% of participants indicating they had been diagnosed in the past. With respect to access to care, 23.9% had received a mental health treatment, while 20.4% looked for treatment but could not access it.

**Table 4:** Mental health and suicidality of trans women participants and correlates of psychological distress, suicidal ideation and suicide attempt (N = 763)

Variables	Descriptive Frequency		Bivariate Models			
			K10 Score		Ideation/Attempt	
	N	%	Estimate	95%CI	Estimate	95%CI
Access to treatment for any mental health condition						
No, but did not look for treatment	425	55.7	1	–	1	–
No and did look for treatment	156	20.4	2.73	1.881–3.999	2.71	1.919–3.826
Yes	182	23.9	1.39	0.973–1.98	2.52	1.814–3.497
Depression diagnosis	146	19.1	3.01	2.08–4.408	4.27	3.003–6.121
Post-traumatic stress disorder diagnosis	41	5.4	1.83	0.972–3.49	5.5	2.848–11.34
Anxiety diagnosis	202	26.5	1.94	1.406–2.695	2.26	1.668–3.065
Psychological distress (based on K10 results)						
None	120	15.7			1	–
Mild	323	42.3			2.27	1.485–3.529
Moderate	252	33			4.27	2.755–6.707
Severe	68	8.9			8.64	4.76–15.993
Suicidal ideation in lifetime	191	25				
Suicide attempt in lifetime	238	31.2				

### Psychological distress

**Fig. 1:**
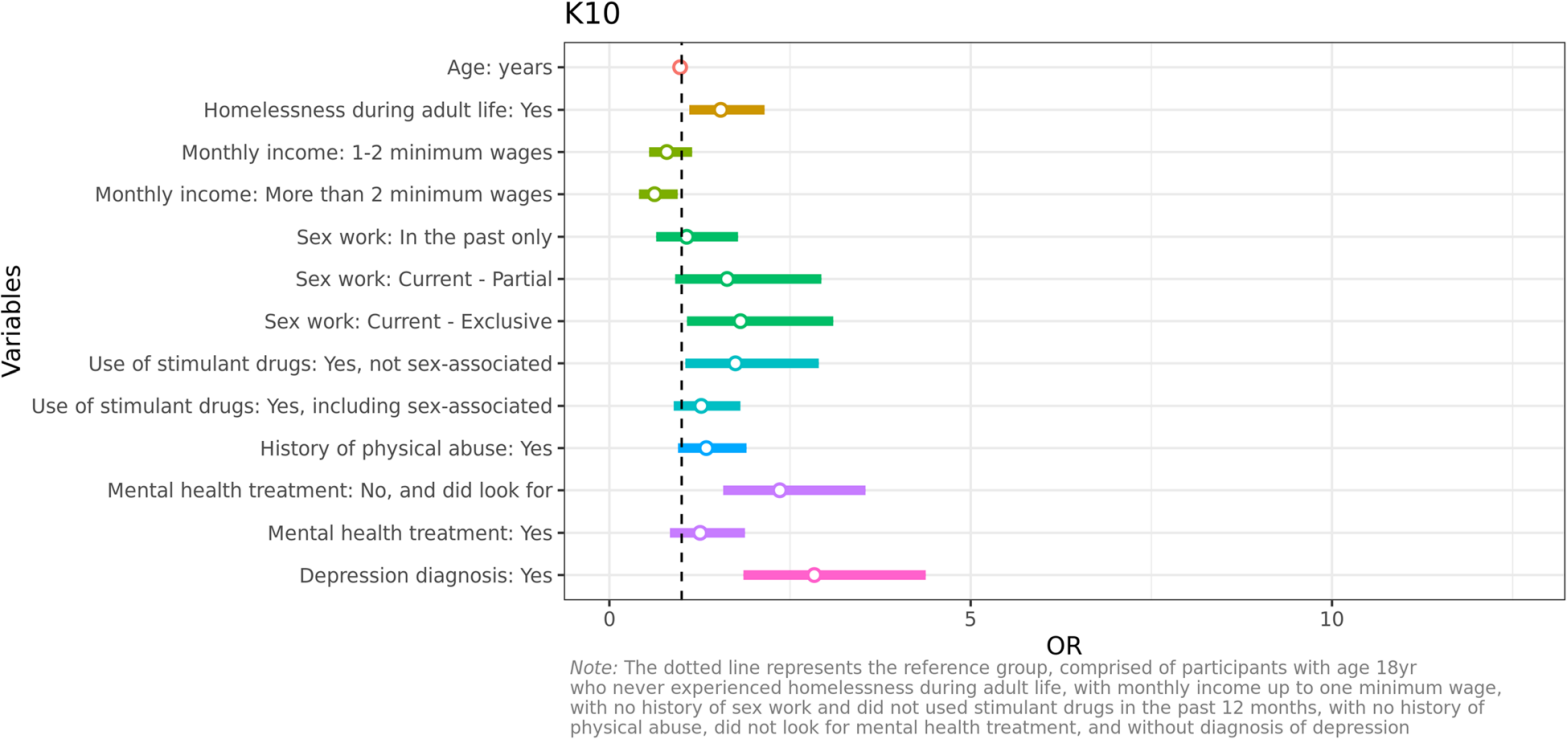
Multivariate logistic model effects on the risk of moderate/high distress

When using the 10-item Kessler scale to establish the level of psychological distress of participants, we found that more than two in five trans women had moderate to severe psychological distress (41.9, 95%CI: 36.6–47.4) (Table 4). Results of the bivariate analysis are presented in Tables 1, 2, 3 and 4. Further, the multiple logistic regression model identified a number of factors that, combined, are associated with psychological distress (Fig. 1). Trans women who have experienced homelessness in adulthood were more likely to experience high levels of psychological distress (OR:1.5, 95%CI:1.09–2.12). Income, on the other hand, was found to be a protective factor, with higher levels of income being associated with lower levels of psychological distress (OR:0.62, 95%CI:0.41–0.94). Currently working as a sex worker was associated with an increase in psychological distress, regardless of whether they are doing it as their main source of income (OR:1.80, 95%CI:1.06–3.08) or only as a partial source (OR:1.63, 95%CI:0.91–2.94), being more likely to experience moderate and severe psychological distress than those who never worked as a sex worker. Using stimulant drugs such as crack and cocaine at times other than before or during sex increases the likelihood of experiencing high levels of psychological distress (OR:1.75, 95%CI:1.05–2.90). Those who additionally use these drugs before or during sex also present an increased risk of psychological distress, although to a lesser extent (OR:1.27, 95%CI:0.89–1.25). Trans women who looked for but could not get access to treatment for mental health conditions were more likely to experience psychological distress than those who had not looked for treatment (OR:2.35, 95%CI:1.5–3.53). Those who looked for and had access to treatment also had an increased risk of psychological distress but to a lesser extent (OR:1.24, 95%CI:0.82–1.86). A depression diagnosis was also significantly associated to psychological distress (OR:2.87, 95%CI:1.87–4.43). Finally, the experience of physical abuse presented a strong trend towards an association with higher levels of psychological distress (OR:1.33, 95%CI:0.94–1.88).

It is important to note that when considering the profile represented by the reference categories, that is, the best possible outcome profile, the probability of presenting high distress is 9.6%. Conversely, when all risk factors are combined, the probability of high levels of psychological distress rises to 96%, ten times higher than the best outcome profile.

### Suicidal ideation and suicide attempts

**Fig. 2:**
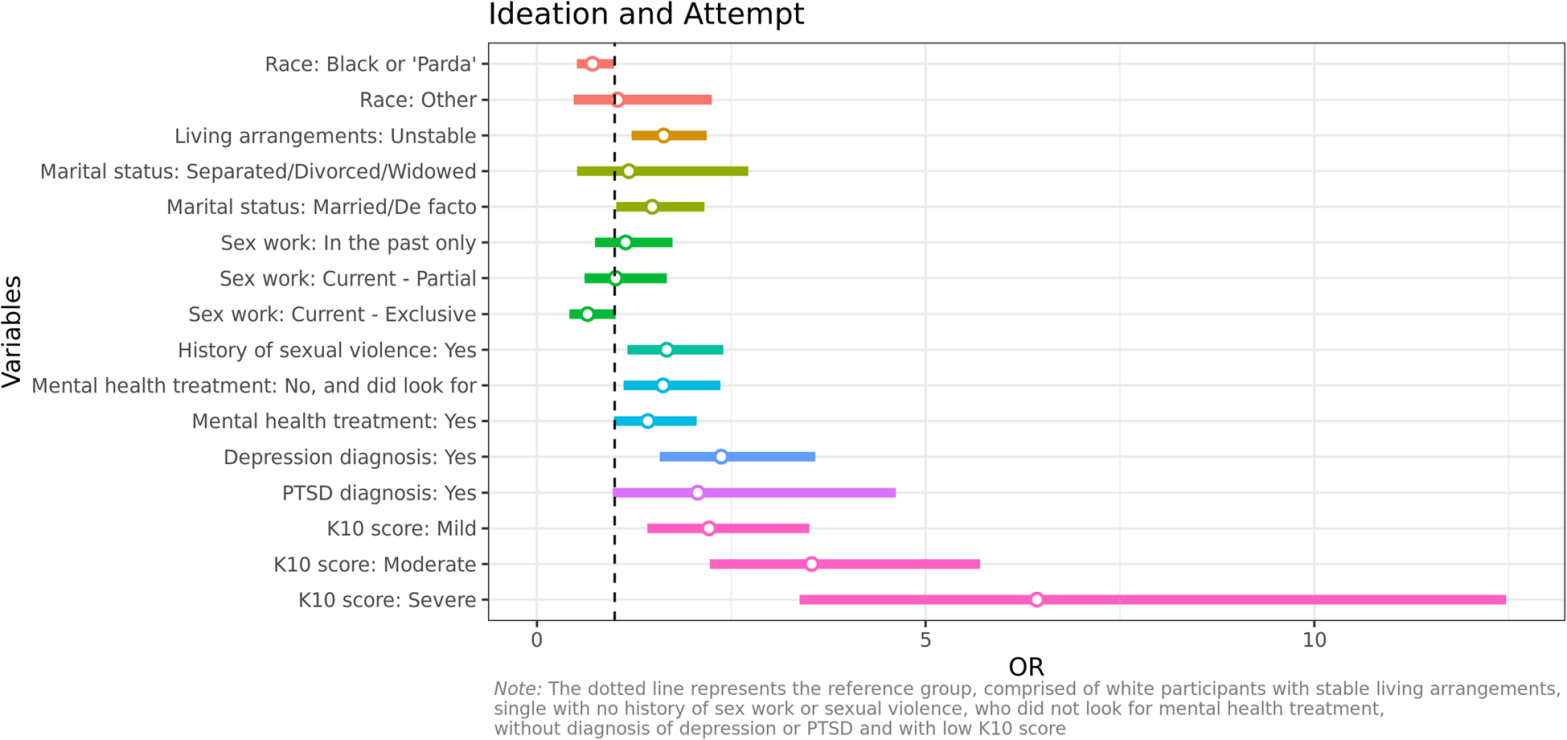
Multivariate ordinal logistic model effects on the risk of suicidal ideation and suicide attempt

More than half of all participating trans women reported having previously either experienced suicidal ideation (25, 95%CI: 18.8–31.2) or attempted to take their own lives (31.2, 95%CI: 25.2–37.0). Results of the bivariate analysis are presented in Tables 1, 2, 3 and 4. Further, the multivariate logistic regression analysis identified a number of factors associated with suicidal ideation and suicide attempt (Fig. 2). A strong association was found between previous diagnosis of depression (OR:2.33, 95%CI:1.55–3.52), and a noteworthy trend towards an increased risk of suicidal ideation and suicide attempt was found for those who had been previously diagnosed with PTSD (OR:2.05, 95%CI:0.96–4.58). Psychological distress was also associated with suicidal ideation and suicide attempt, showing an upward trend the more severe the distress experienced (K10 Mild OR:2.22, 95%CI:1.42–3.52; K10 Moderate OR:3.57, 95%CI:2.25–5.77; K10 Severe OR:6.45, 95%CI:3.38–12.51). Access to treatment for mental health was also associated with suicidal ideation and suicide attempt, with both those who sought but were unable to secure access to treatment (OR:1.62, 95%CI:1.11–2.36) and those who sought and received treatment at higher risk of suicidal ideation and suicide attempt (OR:1.47, 95%CI:1.02–2.12). Experience of sexual violence was significantly associated with suicidal ideation and suicide attempt (OR:1.69, 95%CI:1.18–2.44). Living in an unstable condition (OR:1.63, 95%CI:1.22–2.19) and being married (OR:1.49, 95%CI:1.02–2.17) were also significantly associated with higher risk of suicidal ideation and suicide attempt. Being a sex worker full-time was negatively associated with suicidal ideation and suicide attempt (OR:0.65, 95%CI:0.41–1.01), as well as identifying themselves as Black or Parda (mixed race) (OR:0.72, 95% CI:0.51–1.00).

An analysis of the combined results from the multivariate analysis allows for the identification of the best and the worst outcome profiles, or the profile with the best or worst prognosis for ideation and suicide attempt. The profile with the highest risk for suicide attempt comprises trans women from a race/skin color other than Black/Parda, living in unstable conditions, married, with a history of engaging in sex work (but not currently), a history of sexual violence, depression and PTSD, who have not had access to mental health treatment despite looking for it, and presenting a high (severe) score in the K10 questionnaire. This profile has a 96.1% probability of presenting at least one suicide attempt during their lifetime. The lowest risk profile for suicide attempt or ideation comprises people who identify as Black or Parda, with stable living arrangements, single, engaging in sex work as their only source of income, without a history of sexual violence, depression or PSTD, who did not look for mental health treatment and presenting a low K10 score. This profile has an 85.9% probability of not presenting a suicide ideation or attempt in their lifetime.

## Discussion

Overall, the study results confirm previous studies that have found a high prevalence of psychological distress, suicidal ideation and suicide attempts among trans women [12, 14, 23]. The prevalence of diagnoses of depression, anxiety and post-traumatic stress disorders were comparatively lower than previous international research with transgender people, with studies reporting a prevalence of up to 50% of depression and 37.2% of anxiety [14] among trans women, while our participants reported considerably lower figures (19.1 and 26.5% respectively). This marked difference may be due to lack of access to mental health services and therefore a lack of diagnoses – as indicated previously, government expenditure in mental health services in Brazil is extremely low [7]. Significantly, the multivariate logistic regression analysis showed that those who indicated that they had looked for mental health treatment but were not able to access it presented significantly higher levels of psychological distress. Previous studies have found that trans and gender diverse people experience high levels of discrimination in the public health system in Brazil [33, 34], which may be contributing to this lower prevalence because of healthcare avoidance.

Notwithstanding this apparent lower prevalence of mental health conditions among our sample, when contrasted with data from Brazil, the results become more striking. According to the World Health Organization’s [35] global health estimates, the prevalence of depression and anxiety disorders in the Brazilian population is of 5.8 and 9.3% respectively, which suggests that the possibly under-diagnosed figures in our sample still represent a likelihood of 2.8 to 3.3 times higher for trans women to experience anxiety or depression than the average adult population in Brazil. Significantly, our findings suggest a strong association between depression, PTSD diagnoses and high levels of psychological distress with higher risks of suicidal ideation and suicide attempts.

The socioeconomic characteristics of trans women in São Paulo are also notably associated with general characteristics of disadvantage that are commonly associated with poor mental health outcomes [36]: most are Black or Pardas, presenting very low levels of education and income. Interestingly, however, being Black or Parda was a protective factor for suicidal ideation and suicide attempt, and did not present an association with higher levels of psychological distress in our sample. We speculate that this may be a result of an identity of power that is associated with resilience to adverse circumstances. The number of people self-declaring as Black or Parda in Brazilian census data has been increasing for the past 9 years [37], which may be the result of an increase in political positioning related to race awareness. This may explain the potential resilience demonstrated by Black and Parda participants. More research exploring how intersectionality may affect trans women’s mental health outcomes will help create a better understanding of the experiences of this diverse group of women in our society [38].

Lower education levels were also not associated with higher levels of psychological distress or with suicidality in our sample, but income and homelessness were. This suggests that higher income and housing stability play an important role in supporting trans women’s mental health and may act to prevent suicidal ideation and suicide attempts in this group of women regardless of their education levels.

Most trans women in our sample had been a sex worker in the past or are still currently engaged in sex work. Previous studies have suggested that sex work may exacerbate trans women’s vulnerability to poor mental health outcomes [39, 40]. Given current engagement in sex work was found to be associated with higher levels of psychological distress, it is reasonable to assume, as others have also found [12, 14], that a high proportion of trans women will experience moderate and severe levels of psychological distress at some stage in their lives. However, engaging in sex work currently as a full-time activity and main source of income was a protective factor for suicidal ideation and suicide attempt. The reason for this is not clear but may be explained by the fact that the question about suicidal ideation and suicide attempt referred to any occurrences in their lifetime and we are not able therefore to identify when in their history of sex work the suicidal ideation and suicide attempt might have occurred. Alternatively, sex work may have afforded some trans women with income and housing stability that mediated occupational risks.

As briefly mentioned before, findings suggest alarmingly high levels of experiences of abuse and violence reported by trans women in São Paulo – more than two thirds having experienced either physical or sexual violence in the past, with almost 90% having experienced verbal abuse. Although not an uncommon finding among studies of trans women across the world [22, 41], and somewhat unsurprising given the position of Brazil as the country with the highest rate of trans homicide in the world with a staggering 3664 reported murders from 2008 to 2020 [24], it is still noteworthy and found to be significantly associated with suicidal ideation and suicide attempt.

Previous research on risk factors for suicidality have frequently found that being married or being in a stable relationship was a protective factor for suicidal ideation and suicide attempt [42, 43]. The common rationale is that marriage provides a social, emotional and economic support basis for the couple. However, for trans women in our sample, this does not seem to be the case. Being married or in a de facto relationship was not associated with higher levels of psychological distress but was significantly associated with an increased risk of suicidal ideation and suicide attempts. This is likely a consequence of trans women being particularly vulnerable to being victims of abusive relationships [41]. Previous research has indicated that partners of trans women commonly use coercive strategies taking advantage of trans women’s fears of discrimination and stigma, and may become aggressive when they are threatened with being exposed as a partner of a trans woman [16]. In addition, trans women frequently refrain from reporting partner violence for fear of abuse and discrimination by the police or others [44].

Finally, the findings of the current study point to a concerning high risk of suicide attempt among trans women of a particular profile – for those who identify as being of ‘other’ race, are married and living in unstable conditions, who were sex workers in the past but who are not currently, have a history of sexual violence, depression and PTSD, present severe psychological distress and have sought mental health treatment but were not able to access it, the risk of a suicide attempt is 96.1%, highlighting areas where action can be taken to prevent suicidality. Of note, in times like a global pandemic, only having sex work for income has created serious threats to survival for trans women [45].

These results point to the need for a structural reform in Brazil that enables a reduction in the social inequality and violence that affect the mental health of trans women. Although empirical findings do not necessarily translate into actual policies, evidence-based policies have been actively sought by health professionals, managers and policy makers. In the field of suicide prevention such discussion has been pivotal not only from a public health perspective, but from broader perspectives emerging from the fields of social sciences and economics [46]. The combination of sound data and permanent advocacy targeting the most stigmatized segments of the society is key, especially in the context of policies with strong homo- and trans- phobic components [47].

A number of interventions are recommended based on our findings. First, gender-affirming mental health care available in the public health system that includes intimate partner violence screening is urgently required. Initiatives such as crisis hotlines and support groups have been shown to be effective in suicide prevention broadly [48, 49] and with gender diverse people in particular [50]. In times post-COVID-19, telehealth for mental health counselling should be considered to improve reach and accessibility of services, particularly to those who already experience access barriers. In addition, it is necessary to implement public health programs that include the training of professionals and managers to address issues related to gender identity and consider the impact of factors such as family exclusion, violence, access to education and employability on the health of trans people. There needs to be programs available to combat transphobia in health services, schools and society, and it is essential to promote and expand trans women’s access to mental and general health services. Lastly, housing, employment rights and workforce development to serve trans women is of ultimate importance if we are to be successful in addressing the alarming rates of mental health conditions and suicidality among this marginalized population.

We suggest that further research in this field should connect and engage with the young trans population to identify experiences that can lead to suicidal ideation or suicide attempts. We also suggest that future research implement and evaluate strategies for mental health care and suicide prevention among transgender people.

### Limitations

An important limitation of the present study is that the outcome for suicidal ideation and suicide attempt is a lifetime event and some associated variables had more specific time periods, making direct associations problematic. This limitation notwithstanding, it is crucial to note that previous suicide attempt has been shown to be a strong predictor of a future attempt [51, 52]. Second, as with many studies investigating mental health and suicidality, survivorship bias is a limitation of the present study and may have contributed to an under-representation of the prevalence of suicidal ideation and suicide attempts among trans women. Similarly, the prevalence of mental health conditions such as depression, PTSD and anxiety were based on previous diagnosis, rather than clinical assessments at the time of data collection. As discussed above, access to mental health services is extremely poor in Brazil, and mental health literacy is also lacking, both of which may have contributed to underreporting of mental health conditions. We also acknowledge that trans women experiencing severe depression and anxiety might have received invitations from their peers but chosen not to participate in the study given their mental health conditions, again increasing the potential for underreporting bias. Furthermore, it is important to note that the analysis and results presented here are part of a larger study that was not originally designed to investigate factors associated with mental health conditions, suicide ideation and suicide attempts but focus on HIV incidence and its associated factors among trans gender women. Therefore, there was limited space in the study to explore mental health in depth.

The design and implementation of any scientific study always comprises a deliberate or involuntary trade-off between emphasis on different dimensions of any research such as internal consistency versus generalizability [53]. In our specific case, we invested much of our effort to implement a broad study, in a context where the assessments of such population are scarce and usually limited to clinical-based samples. Nevertheless, this option inevitably compromises the degree specific issues can be explored with the necessary depth. However, despite these limitations, this is the largest published empirical study of trans women’s mental health outcomes and related socioeconomic risk factors in Brazil and, to our knowledge, one of the largest worldwide.

## Conclusions

This study showed that there is a clear association between mental health conditions, lack of treatment for these conditions and suicidality among trans gender women. It also reaffirmed troubling statistics about the dire socioeconomic conditions and high levels of verbal, physical and sexual abuse and violence experienced by trans gender women in Brazil.

## Data Availability

The data that support the findings of this study are available on request from the corresponding author. The data are not publicly available due to privacy or ethical restrictions.
